# Functional characterization of endogenous siRNA target genes in *Caenorhabditis elegans*

**DOI:** 10.1186/1471-2164-9-270

**Published:** 2008-06-03

**Authors:** Suvi Asikainen, Liisa Heikkinen, Garry Wong, Markus Storvik

**Affiliations:** 1 Department of Biosciences, University of Kuopio, P.O. Box 1627, Kuopio 70211, Finland; 2 Department of Neurobiology, A. I. Virtanen Institute, University of Kuopio, P.O. Box 1627, Kuopio 70211, Finland; 3 Department of Pharmacology and Toxicology, University of Kuopio, P.O. Box 1627, Kuopio 70211, Finland

## Abstract

**Background:**

Small interfering RNA (siRNA) molecules mediate sequence specific silencing in RNA interference (RNAi), a gene regulatory phenomenon observed in almost all organisms. Large scale sequencing of small RNA libraries obtained from *C. elegans *has revealed that a broad spectrum of siRNAs is endogenously transcribed from genomic sequences. The biological role and molecular diversity of *C. elegans *endogenous siRNA (endo-siRNA) molecules, nonetheless, remain poorly understood. In order to gain insight into their biological function, we annotated two large libraries of endo-siRNA sequences, identified their cognate targets, and performed gene ontology analysis to identify enriched functional categories.

**Results:**

Systematic trends in categorization of target genes according to the specific length of siRNA sequences were observed: 18- to 22-mer siRNAs were associated with genes required for embryonic development; 23-mers were associated uniquely with post-embryonic development; 24–26-mers were associated with phosphorus metabolism or protein modification. Moreover, we observe that some argonaute related genes associate with siRNAs with multiple reads. Sequence frequency graphs suggest that different lengths of siRNAs share similarities in overall sequence structure: the 5' end begins with G, while the body predominates with U and C.

**Conclusion:**

These results suggest that the lengths of endogenous siRNA molecules are consequential to their biological functions since the gene ontology categories for their cognate mRNA targets vary depending upon their lengths.

## Background

The genome of *C. elegans *contains two principal groups of small RNA species capable of interfering with gene expression. The first, microRNAs (miRNAs), are a class of relatively well characterized small RNAs of ~22 nucleotides (nt) in length derived from a hairpin precursor of ~65–70 nt, that regulate gene expression patterns during organism development and are found from almost all eukaryotes [1,2]. miRNAs are often derived from their own transcript or from intron sequences of protein coding genes [2,3]. Endogenous small interfering RNAs (endo-siRNAs) are a second class of endogenous regulators of gene expression. They are often derived from exons and match perfectly with mRNA sequence of a target gene [3-5]. In contrast, exogenous small interfering RNAs (exo-siRNAs) can be processed from a double stranded RNA (dsRNA) precursor derived from cellular transfections, microinjections, feeding, or from genetic material of invading viruses [7-10]. The biogenesis of exo-siRNAs appears to differ from endo-siRNAs and thus separate pathways for processing and mediating their silencing actions have been proposed [4,5].

According to the current model of siRNA biogenesis, both exogenous and endogenous siRNAs are processed from double-stranded RNA (dsRNA) precursors [7] by Dicer, a ribonuclease III enzyme (RNase III) that cleaves the long double-stranded RNA molecule to yield 21–25 nt siRNAs [2,4]. Subsequent loading of the siRNA into the RNAi silencing complex, followed by action of RNA-dependent RNA polymerase (RdRP) on target mRNA template, yields a population of secondary siRNAs that are able to interfere with gene expression through transcriptional repression, translational block, or mRNA cleavage [2,4,11-13]. RRF-3 is the first identified *C. elegans *RdRP homolog required for accumulation of at least a portion of endo-siRNAs [4,5]. *rrf-3 *mutants lack many endo-siRNAs and have an enhanced RNAi phenotype presumably due to release of it's associated pathway proteins from endogenous RNAi (endo-RNAi) to the exogenous RNAi pathway (exo-RNAi) [4,5,14]. Microarray expression analysis of *rrf-3 *mutants have suggested that endo-siRNAs produced from RRF-3 dependent synthesis regulate a large number of protein coding genes, especially those involved in spermatogenesis and protein phosphorylation [5,15].

Two studies that performed large scale sequencing of small RNA libraries cloned from mixed stage populations of *C. elegans *have provided initial material for grouping and classification of candidate endo-siRNAs [5,6]. From a preliminary analysis, the existence of a sub-class of endo-siRNAs, referred to as 21U-RNAs, have been discovered [6]. 21U-RNAs are precisely 21 nt long, begin with uridine 5'-monophosphate and originate from more than 5700, primarily non-coding, genomic loci which are dispersed in two broad regions of *C. elegans *chromosome IV. Absence of complementary mRNA matches suggests that this siRNA type does not operate by a "classical" mRNA degradation manner of RNAi but may direct alternative actions such as modifications in chromatin structure [6]. Another less abundant class of endo-siRNA that arises from non-coding genomic regions has also been described. These candidate siRNA sequences are dispersed all along the genome and are referred to as tiny non-coding RNAs (tncRNAs) [5]. The absence of corresponding mRNA sequence suggests that tncRNAs may function in a manner similar to 21U-RNAs. Both studies [5,6] observed a number of candidate siRNA sequences perfectly corresponding to one or several mRNA sequences. The mRNA match provides strong evidence that this particular class of endo-siRNA is capable of hybridizing with the gene product and interfering with gene expression by a "classical" mRNA degradation manner.

In order to understand the cellular functions regulated by endogenous siRNAs collected from *C. elegans*, we merged the libraries from two sequencing projects [5,6] containing all siRNA sequences currently available, resulting in a collection of 7136 candidate endo-siRNA sequences. We characterized their length distributions and the relationship of the siRNA with the function of genes targeted. We observed that different lengths of endo-siRNA molecules are associated with functionally different target genes. Moreover, we observe that some argonaute-related genes associate with siRNAs with multiple reads.

## Results

### Length distribution of candidate siRNA sequences

The large libraries of candidate endo-siRNA sequences obtained by sequencing efforts of Lee and co-workers from the Ambros laboratory, and Ruby and co-workers from the Bartel laboratory [5,6] prompted us to merge the libraries and analyze them by length distribution. From the total of 7136 short RNA sequences, 4024 exhibited antisense complementarity to 2344 known mRNA sequences (Additional files 1 and 2). These putative siRNAs were derived from exon coding areas and corresponding mRNAs were defined as cognate targets.

**Figure 1:**
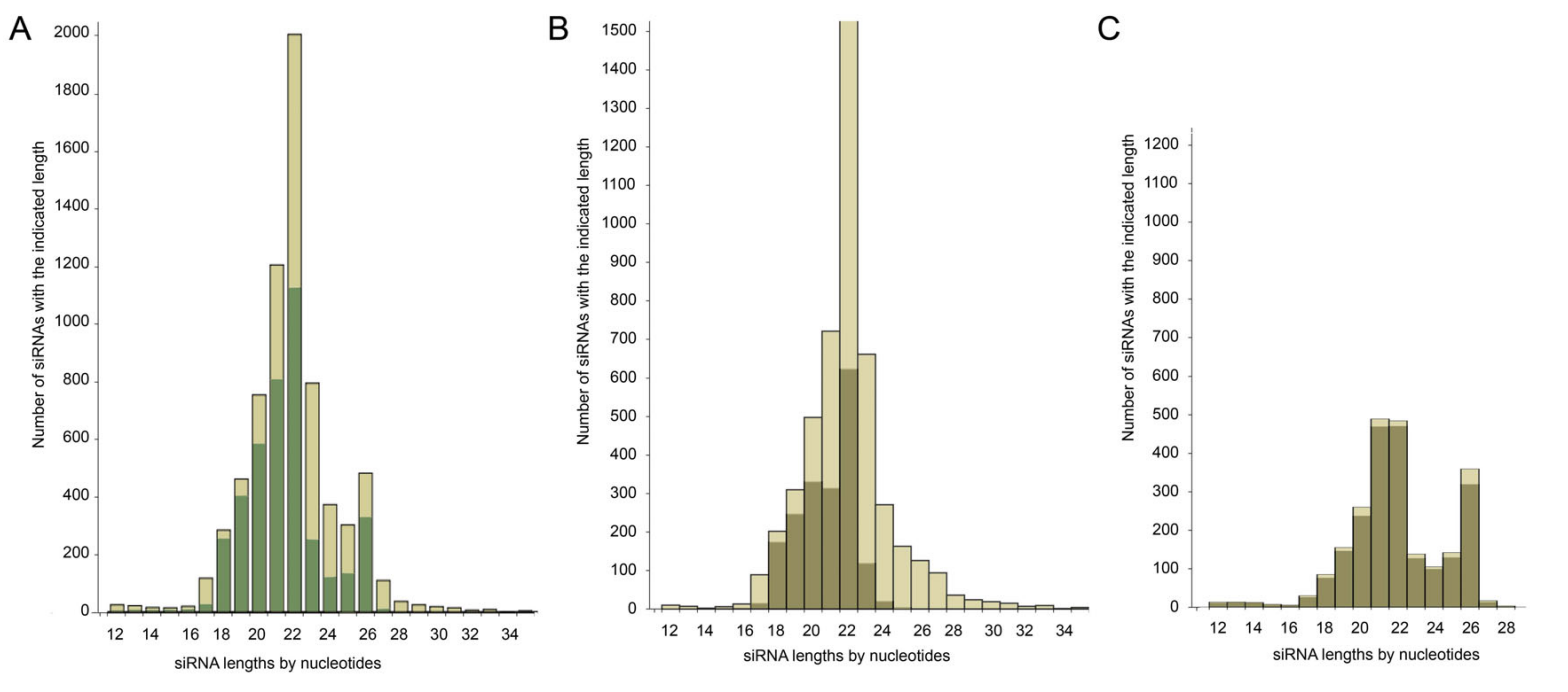
**Length distribution of siRNAs**. The siRNAs were obtained by high-throughput sequencing of mixed stage *C. elegans *populations from two library sources (Lee et al., 2006; Ruby et al., 2006). The height of the bars indicates the number of siRNAs for the respective length. The shaded portion of the histograms indicates the number of siRNAs with an mRNA sequence match. A) Length distribution of all 7136 available short RNA sequences from *C. elegans *available from the two library sources. The length of sequences ranged from 12- to 37 nucleotides. A total of 4024 siRNA sequences matched with an mRNA sequence from the two library sources. B) Length distribution of siRNAs from the library reported in Lee et al., 2006. C) Length distribution of siRNAs from the library reported in Ruby et al., 2006.

Length distributions encompassing all siRNA sequences from the two source libraries are shown in Figure 1a. siRNAs from Lee and co-workers are shown in Figure 1b and siRNAs from Ruby and co-workers are shown in Figure 1c. siRNAs with a mRNA match are shaded and siRNAs without a match are shown in lighter color. In both libraries, the short RNAs not matching with mRNA are widely distributed in length while siRNAs with a match exhibit a more centered distribution. Strikingly, siRNAs with no mRNA match are dramatically overrepresented in the library of Lee and co-workers, while in the library of Ruby and co-workers, the majority of siRNAs match with mRNA. In both libraries, the length distribution of siRNAs matching with mRNA shows a normal distribution arising from a length of 18- to 24-mers with the highest peak at 22-mers (Figure 1b and 1c). A difference arises from the existence of additional 24- to 26-mers in the library of Ruby and co-workers indicating selectivity in the cloning method for these specific lengths of small RNA molecules. In addition, 21-mers are underrepresented in the library of Lee and co-workers when compared with the amount of 21-mers in the library of Ruby and co-workers.

### siRNA sequences with multiple reads

**Figure 2:**
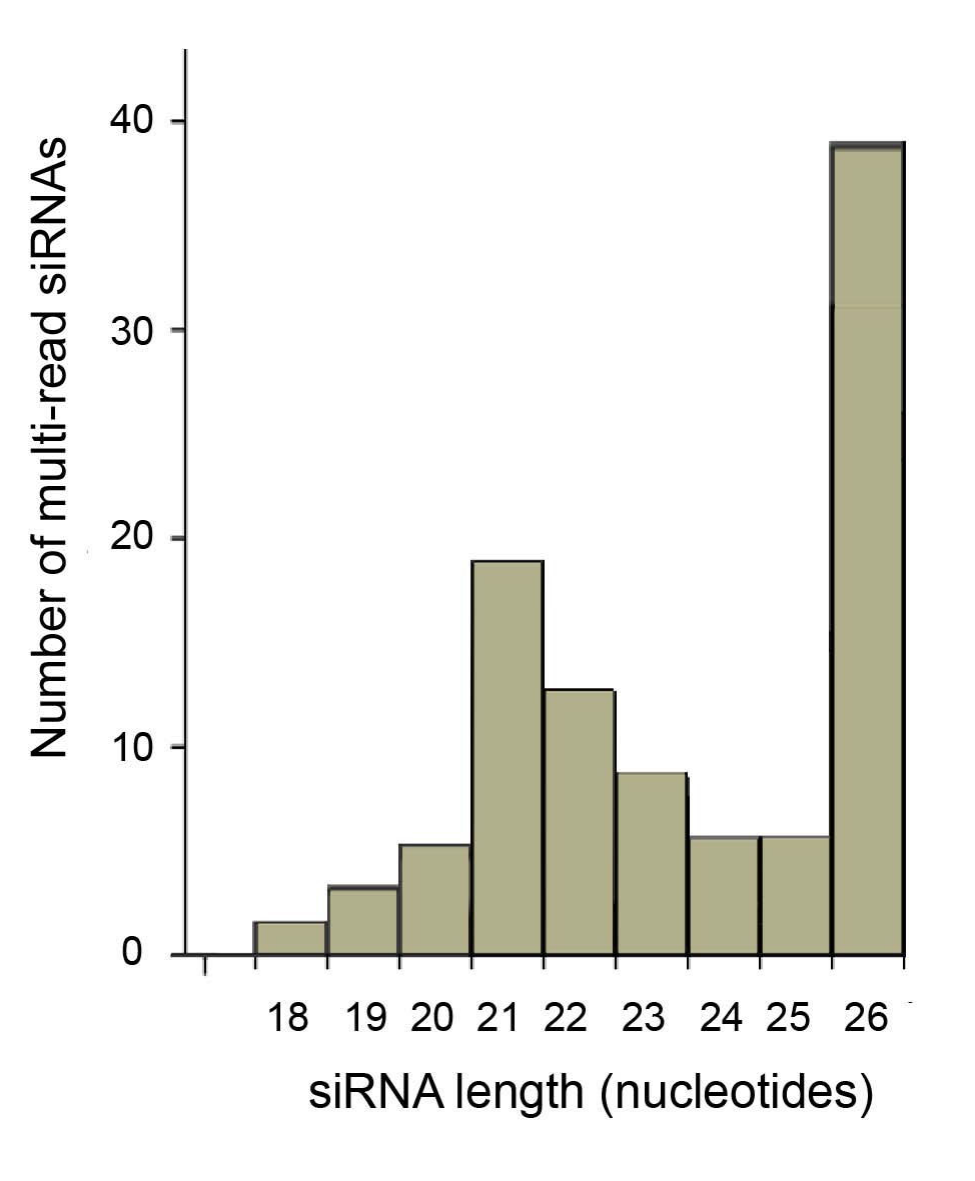
**Length distribution of the multi-read siRNAs**. Multi-read siRNAs were obtained from the library in Ruby et al., 2006 as described in the methods section. The lengths were calculated and the number of counts for each nucleotide length was plotted.

**Figure 3:**
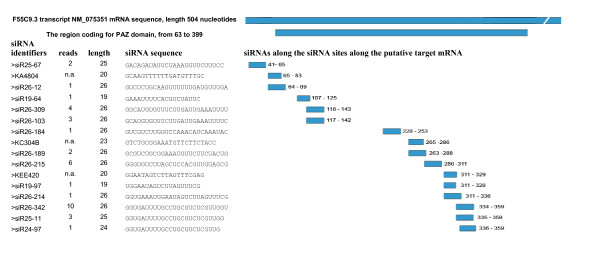
**Length distribution and target sites of siRNAs complementary to Argonaute-related mRNA NM_075351 (F55C9.3)**. The siRNA identifier, number of reads, length, siRNA sequence, and position along the transcript is shown. The short bars indicate the position along the transcript and the numbers indicate the nucleotide position along the sequence. There are one to two nucleotide shorter versions of certain siRNAs that have been read multiple times. The number of reads was not available (n.a.) in some cases because those siRNAs were obtained from Lee et al. (2006).

The abundance (or counts) of individual reads of siRNA sequences was available only for the data from Ruby and co-workers [6]. A total of 125 siRNA sequences were read three or more times with 102 of these matching with mRNA sequences. A list of candidate target genes with 3 or more identical siRNA reads are shown in Additional file 3. Groups of multi-read siRNAs were further observed to exhibit a trend towards overrepresentation of 26-mers (Figure 2). Upon closer inspection, many of the siRNAs with several reads targeted the same mRNAs. When the functions of these putative targets were identified, the "Argonaute and Dicer protein, PAZ" was observed to be significantly enriched as a nomenclature category. The argonaute-related genes targeted by multi-read siRNAs included C16C10.3 (contains PAZ and PIWI RNA-binding domains), F55C9.3 (PAZ) and F58G1.1 (PAZ/PIWI). F55C9.3 was targeted by 16 siRNAs which fell into two length categories: 19- to 20-mers were represented as a single read, while 25- to 26-mers were represented as one to ten reads. All siRNA sites along the gene F55C9.3 (transcript NM_075351) are presented as an example of the distribution of siRNAs along the sequence of the candidate target mRNA (Figure 3).

### Gene Ontology terms correspond to specific siRNA lengths

The targets for each length category siRNA were associated with the specific Gene Ontology (GO) terms shown in Table 1 and Additional file 4. The 18- to 22-mers were associated with the term embryonic development while 23-mers were uniquely associated with post-embryonic development. Longer siRNAs were linked to other GO terms. The 24- to 26-mer siRNAs were linked to phosphorus metabolism or protein modification.

**Table 1:** The enriched GO terms for target genes of endo-siRNAs.

**All siRNAs with a putative target**			
**Biological process**	**n**	**Per cent**	**p-value**

embryonic development	534	23.9	3.6E-44
multicellular organismal process	729	32.7	7.3E-42
reproduction	397	17.8	2.0E-32
larval development	321	14.4	4.3E-24
growth	388	17.4	6.1E-24
post-embryonic development	339	15.2	7.1E-24
cell division	100	4.5	7.9E-23
embryonic cleavage	68	3.0	9.4E-16
sexual reproduction	170	7.6	2.1E-15
reproductive process	185	8.3	1.2E-14
**Molecular function**	**N**	**Per cent**	**p-value**
nucleotide binding	291	13.0	1.7E-23
purine nucleotide binding	245	11.0	4.5E-16
ribonucleotide binding	235	10.5	4.6E-16
ATP binding	205	9.2	6.1E-15
adenyl ribonucleotide binding	205	9.2	7.6E-15
protein binding	398	17.8	9.7E-15
RNA binding	83	3.7	8.6E-12
nucleic acid binding	295	13.2	6.5E-11
helicase activity	28	1.3	1.0E-5
protein serine/threonine kinase activity	83	3.7	1.9E-5
**22 nucleotides long siRNAs**			
**Biological process**	**N**	**Per cent**	**p-value**
multicellular organismal development	293	33.4	1.1E-21
embryonic development ending in birth or egg hatching	221	25.2	2.7E-20
reproduction	170	19.4	7.5E-16
growth	161	18.4	1.1E-10
cell division	44	5.0	1.4E-10
**23 nucleotides long siRNAs**			
**Biological process**	**N**	**Per cent**	**p-value**
multicellular organismal development	87	25.4	1.6E-5
reproduction	50	14.6	1.9E-4
anatomical structure development	34	9.9	4.9E-4
cell division	14	4.1	7.5E-4
cell cycle process	13	3.8	1.2E-3
**26 nucleotides long siRNAs**			
**Biological process**	**N**	**Per cent**	**p-value**
phosphate metabolic process	26	11.9	4.4E-09
biopolymer modification	27	12.3	1.7E-07
cellular protein metabolic process	32	14.6	1.1E-04
cellular macromolecule metabolic process	32	14.6	2.0E-04
biopolymer metabolic process	38	17.4	2.4E-04

Target gene lists were submitted to DAVID 2.0 as described in methods. The 10 most significant GO categories are shown for all siRNAs with a putative target. The 5 most significant GO categories are presented for the siRNAs according to their length. The lists for siRNAs of all lengths are presented in Additional file 4.

### Nucleotide sequence of siRNA molecules

**Figure 4:**
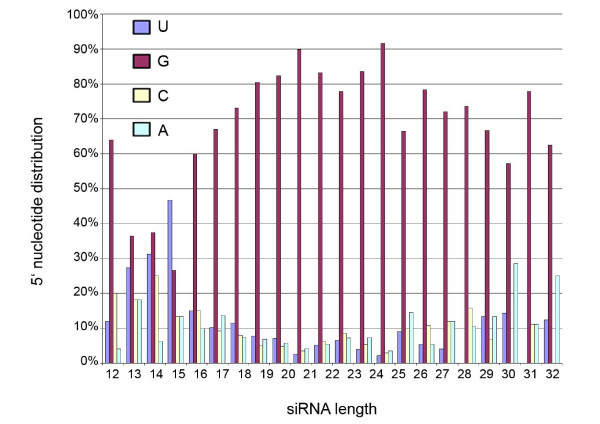
**The distribution of 5' nucleotides in siRNAs grouped by the length**. Each bar represents the percentage of siRNAs for the given length. The shading indicates the 5' starting nucleotide.

**Figure 5:**
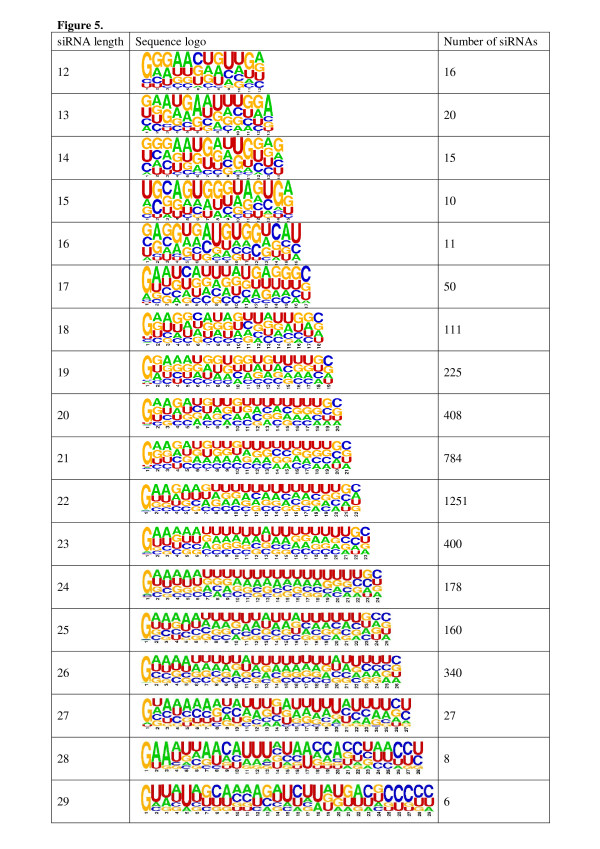
**The frequency sequence graphs (logos) of siRNAs which have putative target mRNAs**. siRNA sequences were collected and divided by size as described in methods. Sequence logos were generated using WebLogo . The siRNA length is shown on the left and the number of siRNAs in each length category is shown on the right.

The starting nucleotide was previously shown to be guanine (G) among 85% of endogenous siRNAs in the library of Ruby and co-workers [6]. Thus, it was of interest to measure the most common starting nucleotide after combining libraries. After plotting the starting nucleotide of siRNA sequences against their length, we observed a similar trend with G as the most abundant first 5' nucleotide (Figure 4). Shorter siRNAs exhibited uracil (U), cytosine (C) or adenine (A) frequently as the starting nucleotide. Furthermore, we constructed frequency sequence graphs (logos) for 4024 siRNA sequences of 12–29 nt in length with an mRNA match (Figure 5). All lengths of siRNAs prefer G in their 5' end and C in their 3' end with both A and U largely represented along the body. All siRNAs from the combined library exhibited an average A/U content of 53.1%, while A/U content of all mRNAs was previously reported to be 42.7% [16] and 64.6% in the whole genome [17]. 12- to 16-mers exhibit variable 3' nucleotides (A, G or U) suggesting that short sequences are potential degradation products of longer sequences. To address this question, we aligned siRNA sequences and observed that 5 of 25 sequences (20%) among 12-mers and 3 of 22 sequences (13.6%) among 13-mers could be found from longer siRNA sequences (Additional file 5). Frequency sequence graphs were generated also for the combined library of 7136 RNA sequences (12- to 33-mers) including those without mRNA matches (Additional file 6). Results were similar between the two sets of frequency sequence graphs.

### Conservation of candidate mRNA target sequences

The conservation of the putative target genes for the siRNAs was inspected by using pre-calculated BLAST similarities between *C. elegans *and *C. briggsae *(Wormbase release WS170). 14.4% of genes had perfect matches between the two species, while only 8.8% of the rest of the genome had this score reflecting conservation of siRNA associated genes (data not shown). However, the genes targeted by several different siRNAs were found to be more weakly conserved than the target genes on average (Additional file 7).

## Discussion

The existence of endogenous siRNAs (endo-siRNAs) encoded by the genome in *C. elegans *has been reported by several groups [2,4-6]. The evidence for classification of these sequences as endo-siRNAs has been the observation that RNAi machinery is required for their accumulation. In addition, many RNAi pathway mutants exhibit elevated levels of gene expression indicating the loss of regulatory RNAs [5,15]. We combined datasets representing candidate endogenous siRNAs collected to date from *C. elegans *by high-throughput sequencing efforts [5,6]. Two electronic libraries of endo-siRNAs were gathered and annotated.

The length of siRNAs in a combined data set appeared to be about 22 nt on average, which was observed for siRNA sequences with either matching or non-matching mRNAs (Figure 1a). Examination of the siRNAs as separate groups by the source laboratory appeared to affect their length distributions with 26-mers more highly represented in the library of Ruby and co-workers [6] (Figure 1c). In both libraries, centered distributions of 18- to 23-mers suggest a uniform class of endogenous siRNAs with ability to hybridize with mRNA sequences (Figures 1a, 1b and 1c). siRNAs with no mRNA matches exhibited a wider length distribution. It is possible that siRNAs without mRNA matches represent a less uniform group suggesting alternative modes of synthesis for these sequences. siRNAs in the library of Ruby and co-workers [6] were enriched for siRNAs with matching mRNAs (Figure 1c) while siRNAs with fewer mRNA matches were more highly represented in the library of Lee and co-workers [5] (Figure 1b). A possible explanation could arise from the characteristics of the cloning method used by the two laboratories. The siRNA-library by Lee and co-workers was constructed using a 5' monophosphate ligation independent cloning manner, while the construction of the library by Ruby and co-workers utilized the linker sequence with bias to detect 5' monophosphate ends of siRNA molecules. The higher proportion of siRNAs with mRNA matches in the library by Ruby and co-workers could be explained through a case where the majority of these sequences captured with the 5' monophosphate arise from secondary siRNA synthesis on the mRNA template. Secondary siRNAs via the exo-RNAi pathway have been shown to prefer a 5' triphosphate end, while primary siRNAs are generally thought to contain 5' monophosphate in the Dicer dependent synthesis [9,12]. However, the 5' monophosphate could also be obtained by the activity of enzymes with ability to remove 5' γ- and β-phosphates such as *C. elegans *PIR-1 [4]. Another possibility to obtain a 5' monophosphate end would be the removal of the entire 5' nucleotide with the triphosphate [4,6,12]. The existence of abundant 25-mers along with even more abundant 26-mers provides support for this hypothesis. The argonaute-related gene F55C9.3 was chosen to model the distribution of siRNA sequences along the mRNA. A total of 16 unique siRNAs with 1–10 reads each, aligned on the F55C9.3 mRNA fall into two interesting length categories. The length difference between 19- to 20-mers and 24- to 26-mers reflects the existence of two individual classes of siRNAs on the mRNA. It is tempting to speculate that the shorter ones with only one read exhibit members of a class of primary siRNAs and the abundant longer ones with up to ten identical reads as secondary siRNAs synthesized by a RdRP. However, biochemical studies are needed to further characterize these classes of siRNAs.

Since the siRNAs from the two sources available so far have over 2200 candidate target genes in total, we categorized these genes by their Gene Ontologies (Table 1, and additional file 4). An interesting observation was that the lengths of endogenous siRNAs seem to determine the functional characterization of their putative target genes. The matching mRNAs for 22-mer siRNAs were associated with the GO term embryonic development while candidate targets for 23-mers were uniquely associated with post-embryonic development. It has been shown that some endogenous siRNAs and almost all miRNAs exhibit developmentally regulated expression patterns [1,2]. Interestingly, the 24- to 26-mer siRNAs were associated with phosphorus metabolism or protein modification. We hypothesize that the synthesis of these relatively long 25- to 26-mer endo-siRNA molecules could occur by specific RdRP such as RRF-3 and its associated proteins since a large group of phosphorus metabolism linked genes was observed to be over-expressed in *rrf*-3 mutant worm strains [15]. RRF-3 has shown to be required for the biogenesis of several classes of endogenous secondary siRNAs [4,5]. In addition, the *C. elegans *argonaute family of RNA binding proteins could exhibit specificity for the length of siRNA and direct silencing of genes associated with specific biological processes such as embryonic development, post-embryonic development, or phosphorus metabolism.

After plotting the starting nucleotide of siRNA sequences against their length, we observed that most siRNAs have G at their 5' end. In addition, sequence frequency graphs showed preference for C at their 3' end. A and U were largely represented along the body of siRNA molecules. A/U frequency might be needed to lower the energy required to remove newly synthesized endo-siRNA molecules and allow the RdRP complex to continue unprimed production of additional secondary siRNA molecules along the template [12,18]. Only short siRNAs of 12 to 16 nt in length exhibited variable 3' nucleotides (A, G or U) suggesting that these are potential degradation products of longer ones.

Target genes for the siRNAs were observed to be more highly conserved between *C. elegans *and *C. briggsae *than the genes on average. One explanation is that siRNAs target genes with conserved exons. It is also possible that evolutionary conserved target genes have had time to develop siRNA sequences, for example, to regulate their expression during specific developmental stages. When each target gene was classified by the number of associated siRNAs, it was observed that the target genes with more than three siRNAs tended to be poorly conserved between the two nematode species. This might indicate the accelerated production of secondary siRNAs for young species specific genes.

## Conclusion

In order to understand the cellular functions regulated by endogenous siRNAs collected to date from *C. elegans*, we merged the libraries from two sequencing projects [5,6] containing all publicly available siRNA sequences resulting in a collection of 7136 endo-siRNA sequences. We characterized their length distributions and the relationship of the siRNA length with the function of genes targeted. The endo-siRNA sequences corresponded to functionally different target genes that were dependent upon the siRNA length: 18- to 22-mers match mRNA targets associated with embryonic development, targets of 23-mer siRNAs associate with post-embryonic development and targets corresponding to 24–26-mer siRNAs involve phosphorus metabolism or protein modification. Genes targeted with siRNAs with multiple reads included several poorly characterized argonaute-related genes. We conclude that the function of target genes of endogenous siRNAs appear to vary depending upon their length. These results also provide additional evidence for existence of a number of siRNA biosynthesis mechanisms capable of regulating gene expression associated with specific biological processes.

## Methods

### Sequences

The siRNA sequences obtained from mixed stage *C. elegans *populations by high-throughput sequencing technologies were obtained from previously published studies [5,6]. Lists of 7136 siRNA sequences in total were annotated by using the NCBI BLAST server with Wormbase release WS170 nematode RefSeq mRNA database, which contains only high-quality sequences for gene transcripts. The results were filtered to contain only plus/minus hits against the known mRNAs representing the antisense siRNA sequences. The results were obtained as RefSeq mRNA identifiers that were unique to known transcripts.

### Length distributions

siRNAs with equal sequence lengths were grouped together and the sequence frequency graphs were generated separately. Nucleotide frequencies of the included siRNAs were created using WebLogo (University of Berkeley, CA, USA). The same process was repeated for the subset of siRNAs that were associated with mRNA sequences.

### Functional categories

The enriched functional categories for the siRNAs with different lengths were obtained using David 2.0 web service [19]. The descriptions for the genes that were targets of 8 or more siRNAs were obtained using Biomart together with WB180 genome. In order to study the conservation of the genes coding the putative target mRNAs, the precalculated BLAST expectation (E) values for the observed homologies between the putative target genes in *C. elegans*, and their orthologs in *C. briggsae*, were obtained using Wormbase WB180. SPSS version 14 was used in the graphical presentation of the results (SPSS Inc., Chicago, Illinois, USA).

## Authors' contributions

SA conceptualized, planned, performed and designed the analysis and wrote the manuscript. LH performed the experiments and participated in writing the manuscript. GW participated in the design of the experiment, management of the project, and writing of the manuscript. MS performed the experiments, wrote the manuscript, and managed the data and submission. All authors read and approved the final manuscript.

## Supplementary Material

Additional file 1 **The collection of all 4024 siRNAs with putative mRNA targets**. The sequences, lengths, and the putative target mRNAs for the siRNAs from two sources (Lee et al., 2006, Ruby et al., 2006) are presented. Click here for file

Additional file 2 **The collection of all 7136 short RNAs**. The sequences, lengths, and the 5'nucleotides of the entire RNA collection from two sources (Lee et al., 2006, Ruby et al., 2006) are presented. Click here for file

Additional file 3 **The individual siRNAs with the most reads**. The data from Ruby et al (2006) contained numbers of individual detections of the sequence and they are presented here. Click here for file

Additional file 4 **The full list of the enriched GO terms of siRNAs by length**. Target gene lists were submitted to DAVID 2.0 as described in methods. The 10 most significant GO categories are shown for all siRNAs with a putative target. The 5 most significant GO categories are presented for the siRNAs according to their length. Click here for file

Additional file 5 **Redundancy and fragments of short siRNAs**. The number of short siRNAs that were aligned with longer siRNAs are presented on the first leaf. On the second leaf, the siRNAs (siRNA1) whose sequence is included in one or more longer siRNA sequences (siRNA2) with a perfect match are presented. Click here for file

Additional file 6 **The frequency sequence graphs (logos) of the whole collection of short RNAs**. The sequences included those without putative targets from Lee et al. (2006) and Ruby et al. (2006). The number of siRNAs in each length category is shown on the right. Click here for file

Additional file 7 **Conservation of genes targeted by several siRNAs**. Some genes (mRNAs) were putative targets for many siRNAs. The genes with the highest number of siRNAs appears to be poorly conserved in recent evolution. The homology between the *C. elegans *and *C. briggsae *was estimated by precalculated BLAST Expectation (E)-values. The smaller (down to 10^-300) the E-value, the better statistical value for the homology. Certain genes, such as K02E2.6 did not appear to have an ortholog in *C. briggsae*. Click here for file
