# Supplementary material for: Functional characterization of endogenous siRNA target genes in Caenorhabditis elegans
Source: BMC Genomics. 2008 Jun 3;9:270. doi: 10.1186/1471-2164-9-270 (PMC2440555; doi:10.1186/1471-2164-9-270)
Supplement: Additional file 4 — The full list of the enriched GO terms of siRNAs by length. Target gene lists were submitted to DAVID 2.0 as described in methods. The 10 most significant GO categories are shown for all siRNAs with a putative target. The 5 most significant GO categories are presented for the siRNAs according to their length. [file 1471-2164-9-270-S4.pdf]

Supplementary table 5.

		n	Per cent	p-value
**All siRNAs with putative target**	**Biological process**	**n**	**Per cent**	**p-value**
	embryonic development	534	23.9	3.6E-44
	multicellular organismal process	729	32.7	7.3E-42
	reproduction	397	17.8	2.0E-32
	larval development	321	14.4	4.3E-24
	growth	388	17.4	6.1E-24
	post-embryonic development	339	15.2	7.1E-24
	cell division	100	4.5	7.9E-23
	embryonic cleavage	68	3.0	9.4E-16
	sexual reproduction	170	7.6	2.1E-15
	reproductive process	185	8.3	1.2E-14
	**Molecular function**	**n**	**Per cent**	**p-value**
	nucleotide binding	291	13.0	1.7E-23
	purine nucleotide binding	245	11.0	4.5E-16
	ribonucleotide binding	235	10.5	4.6E-16
	ATP binding	205	9.2	6.1E-15
	adenyl ribonucleotide binding	205	9.2	7.6E-15
	protein binding	398	17.8	9.7E-15
	RNA binding	83	3.7	8.6E-12
	nucleic acid binding	295	13.2	6.5E-11
	helicase activity	28	1.3	1.0E-5
	protein serine/threonine kinase activity	83	3.7	1.9E-5
**18 nucleotides long siRNAs**	**Biological process**	**n**	**Per cent**	**p-value**
	cellular component organization and biogenesis	17	16.2	7.9E-6
	embryonic development ending in birth or egg hatching	28	26.7	5.6E-5
	developmental process	36	34.3	6.0E-5
	multicellular organismal development	35	33.3	7.5E-5
	embryonic development	28	26.7	8.5E-5
	**Molecular function**	**n**	**Per cent**	**p-value**
	purine ribonucleotide binding	15	14.3	4.2E-3
	ribonucleotide binding	15	14.3	4.2E-3
	purine nucleotide binding	15	14.3	6.8E-3
	microtubule motor activity	3	2.9	1.1E-2
	nucleotide binding	15	14.3	1.8E-2
**19 nucleotides long siRNAs**	**Biological process**	**n**	**Per cent**	**p-value**
	cell cycle	17	8.1	2.0E-7
	cell division	16	7.7	9.0E-7
	embryonic development	54	25.8	1.3E-6
	organelle organization and biogenesis	18	8.6	8.7E-6
	microtubule-based process	10	4.8	6.4E-5
	**Molecular function**	**n**	**Per cent**	**p-value**
	nucleic acid binding	31	14.8	6.0E-3
	actin binding	4	1.9	1.6E-2
	enzyme binding	3	1.4	2.7E-2
	nucleotide binding	24	11.5	2.9E-2
	cytoskeletal protein binding	4	1.9	3.6E-2

		n	Per cent	p-value
20 nucleotides long siRNAs	**Biological process**	**n**	**Per cent**	**p-value**
	developmental process	130	37.6	6.4E-12
	embryonic development ending in birth or egg hatching	94	27.2	5.9E-10
	reproduction	76	22.0	2.3E-9
	multicellular organismal process	127	36.7	2.7E-9
	cell division	25	7.2	6.7E-9
	**Molecular function**	**n**	**Per cent**	**p-value**
	nucleic acid binding	61	17.6	3.8E-6
	nucleotide binding	50	14.5	3.0E-5
	RNA binding	16	4.6	1.5E-3
	protein binding	66	19.1	1.6E-3
	adenyl nucleotide binding	36	10.4	1.8E-3
21 nucleotides long siRNAs	**Biological process**	**n**	**Per cent**	**p-value**
	embryonic development ending in birth or egg hatching	193	30.2	4.2E-27
	multicellular organismal development	230	35.9	3.5E-20
	reproduction	139	21.7	2.8E-16
	post-embryonic development	121	18.9	2.6E-13
	larval development	114	17.8	6.3E-13
	**Molecular function**	**n**	**Per cent**	**p-value**
	nucleic acid binding	107	16.7	1.2E-9
	RNA binding	31	4.8	6.7E-7
	protein binding	121	18.9	3.0E-6
	nucleotide binding	80	12.5	7.4E-6
	purine ribonucleotide binding	65	10.2	2.1E-4
22 nucleotides long siRNAs	**Biological process**	**n**	**Per cent**	**p-value**
	multicellular organismal development	293	33.4	1.1E-21
	embryonic development ending in birth or egg hatching	221	25.2	2.7E-20
	reproduction	170	19.4	7.5E-16
	growth	161	18.4	1.1E-10
	cell division	44	5.0	1.4E-10
	**Molecular function**	**n**	**Per cent**	**p-value**
	nucleotide binding	120	13.7	6.8E-10
	nucleic acid binding	138	15.8	3.6E-9
	protein binding	174	19.9	4.0E-9
	purine ribonucleotide binding	98	11.2	2.1E-7
	ATP binding	86	9.8	4.7E-7
23 nucleotides long siRNAs	**Biological process**	**n**	**Per cent**	**p-value**
	multicellular organismal development	87	25.4	1.6E-5
	reproduction	50	14.6	1.9E-4
	anatomical structure development	34	9.9	4.9E-4
	cell division	14	4.1	7.5E-4
	cell cycle process	13	3.8	1.2E-3
	**Molecular function**	**n**	**Per cent**	**p-value**
	protein binding	52	15.2	3.5E-3
	nucleotide binding	33	9.7	1.1E-2
	purine ribonucleotide binding	28	8.2	2.0E-2
	metal ion binding	40	11.7	3.2E-2
	cofactor binding	8	2.3	4.4E-2

	Biological process	n	Per cent	p-value
**24 nucleotides long siRNAs**	**Biological process**	**n**	**Per cent**	**p-value**
	phosphorus metabolism	11	10.7	5.9E-04
	phosphate metabolism	11	10.7	5.9E-04
	biopolymer modification	13	12.6	7.6E-04
	protein modification	12	11.7	2.1E-03
	protein amino acid phosphorylation	8	7.8	3.6E-03
	**Molecular function**	**n**	**Per cent**	**p-value**
	protein serine/threonine kinase activity	7	6.8	8.1E-04
	protein kinase activity	9	8.7	3.3E-03
	phosphotransferase activity. alcohol group as acceptor	9	8.7	6.1E-03
	kinase activity	9	8.7	8.5E-03
	transferase activity. transferring phosphorus-containing groups	9	8.7	2.3E-02
**25 nucleotides long siRNAs**	**Biological process**	**n**	**Per cent**	**p-value**
	biopolymer modification	7	6.6	8.7E-02
	protein modification	7	6.6	7.7E-02
	macromolecule metabolism	12	11.3	7.2E-02
	protein metabolism	10	9.4	7.2E-02
	biopolymer metabolism	10	9.4	3.0E-02
	**Molecular function**	**n**	**Per cent**	**p-value**
	protein serine/threonine kinase activity	11	8.0	1.3E-4
	ATP binding	16	11.7	3.4E-4
	adenyl ribonucleotide binding	16	11.7	3.5E-4
	adenyl nucleotide binding	16	11.7	6.7E-4
	protein kinase activity	11	8.0	8.0E-4
**26 nucleotides long siRNAs**	**Biological process**	**n**	**Per cent**	**p-value**
	phosphate metabolic process	26	11.9	4.4E-09
	biopolymer modification	27	12.3	1.7E-07
	cellular protein metabolic process	32	14.6	1.1E-04
	cellular macromolecule metabolic process	32	14.6	2.0E-04
	biopolymer metabolic process	38	17.4	2.4E-04
	**Molecular function**	**n**	**Per cent**	**p-value**
	protein serine/threonine kinase activity	19	8.7	8.5E-08
	transferase activity, transferring phosphorus-containing groups	22	10.0	9.2E-06
	phosphotransferase activity, alcohol group as acceptor	19	8.7	9.8E-06
	adenyl nucleotide binding	26	11.9	1.3E-05
	ATP binding	24	11.0	4.2E-05

In order to best describe all large functional groups among the putative siRNA targets in the whole collection, the excessive redundancy of GO terms was parsed when observed. For example the similar count for "developmental process", "multicellular organismal development", and "embryonic development" which occurs at different levels of GO hierarchy with nearly identical enrichement (p values 2.8E-49, 9.0E-48, and 3.6E-44, respectively) was presented by the most accurately describing term, i.e. "embryonic development, p value 3.6E-44"
