# Supplementary material for: Functional characterization of endogenous siRNA target genes in Caenorhabditis elegans
Source: BMC Genomics. 2008 Jun 3;9:270. doi: 10.1186/1471-2164-9-270 (PMC2440555; doi:10.1186/1471-2164-9-270)
Supplement: Additional file 6 — The frequency sequence graphs (logos) of the whole collection of short RNAs. The sequences included those without putative targets from Lee et al. (2006) and Ruby et al. (2006). The number of siRNAs in each length category is shown on the right. [file 1471-2164-9-270-S6.pdf]

Supplementary Figure 1: The frequency sequence graphs (logos) of the combined library of short RNAs, also including those without putative targets, from Lee et al. (2006) and Ruby et al. (2006). The number of siRNAs in each length category is shown on the right.

siRNA length	Sequence logo	Number of siRNAs
12	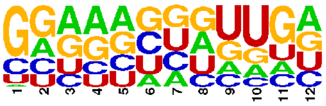	25
13	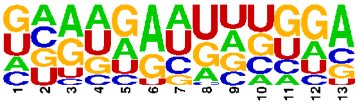	22
14	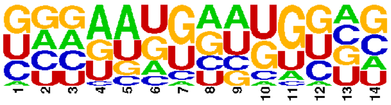	16
15	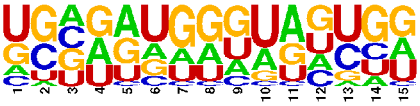	15
16	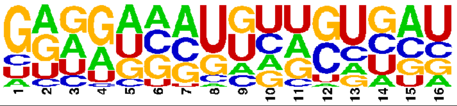	20
17	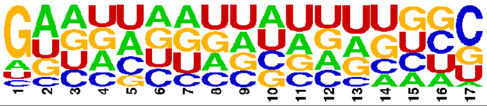	118
18	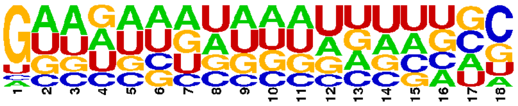	286
19	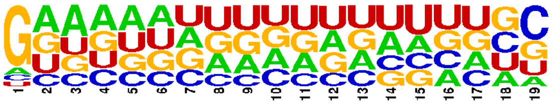	464
20	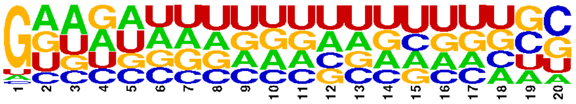	757
21	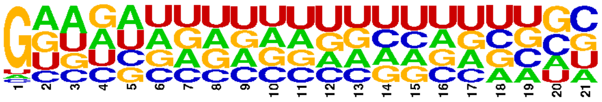	1209
22	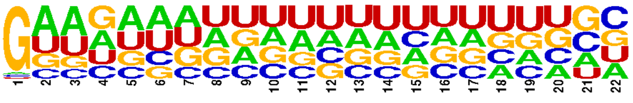	2013
23	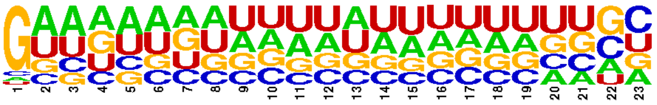	798
24	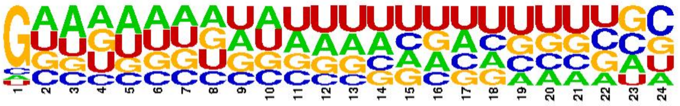	375
25	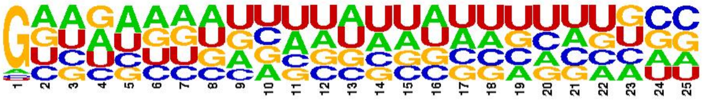	304

26	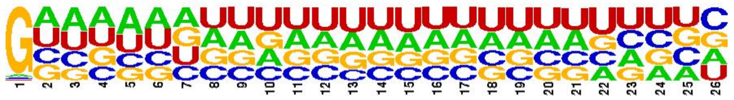	484
27	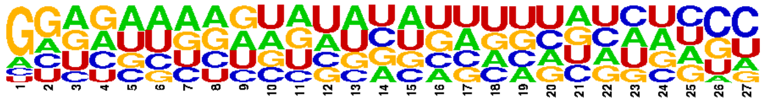	110
28	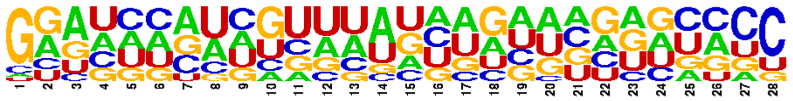	37
29	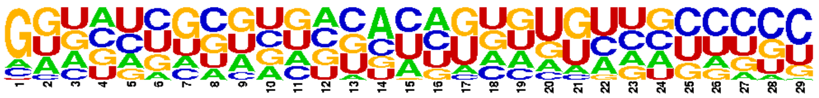	25
30	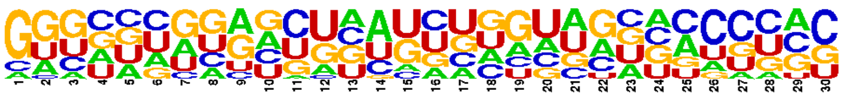	19
31	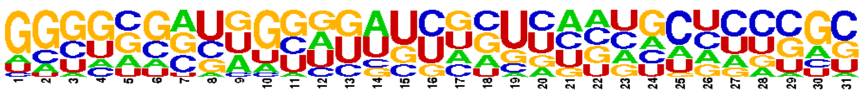	15
32	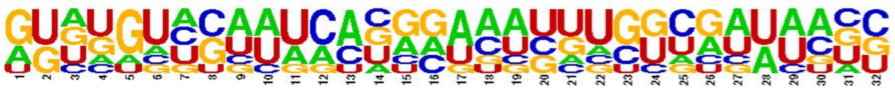	7
33	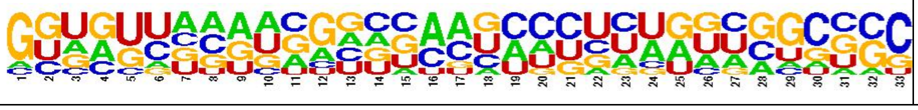	9
